# Prodrugs in Cardiovascular Therapy

**DOI:** 10.3390/molecules13051156

**Published:** 2008-05-14

**Authors:** Marinella G. Sandros, Chady B. Sarraf, Maryam Tabrizian

**Affiliations:** 1 Department of Biomedical Engineering, McGill University, 3775 University Street, Montreal, QC, Canada H3A2B4; 2 Faculty of Dentistry, McGill University, 3640 University Street, Montreal, QC, Canada, H3A 2B2; 3 Center for Biorecognition and Biosensors, McGill Institute for Advanced Materials, 3775 University Street, Montreal, QC, Canada H3A2B4; 4 Department of Medical Education, Seton Hall University, 400 South Orange Avenue, South Orange, NJ 07079, USA; 5 St. Michael’s Medical Center, 111 Central Avenue, Newark, NJ 070102, USA

**Keywords:** Prodrug, cardiovascular disease, drug-eluting stents, antiplatelet, antithrombin

## Abstract

Prodrugs are biologically inactive derivatives of an active drug intended to solve certain problems of the parent drug such as toxicity, instability, minimal solubility and non-targeting capabilities. The majority of drugs for cardiovascular diseases undergo first-pass metabolism, resulting in drug inactivation and generation of toxic metabolites, which makes them appealing targets for prodrug design. Since prodrugs undergo a chemical reaction to form the parent drug once inside the body, this makes them very effective in controlling the release of a variety of compounds to the targeted site. This review will provide the reader with an insight on the latest developments of prodrugs that are available for treating a variety of cardiovascular diseases. In addition, we will focus on several drug delivery methodologies that have merged with the prodrug approach to provide enhanced target specificity and controlled drug release with minimal side effects.

## Introduction

**Figure 1:**
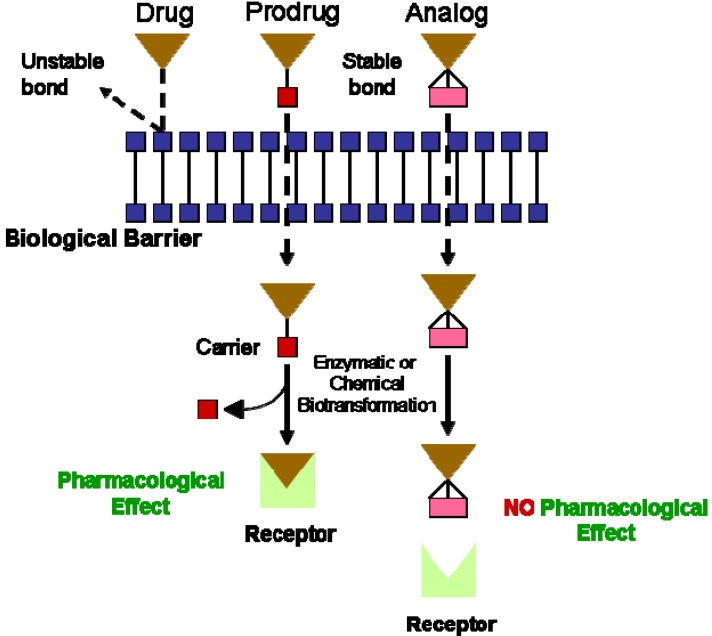
The distinction between prodrugs and analogs (adapted from [2]).

Numerous therapeutic drugs for treating cardiovascular ailments suffer from undesirable properties after metabolism leading to drug inactivation causing pharmacological, pharmaceutical and pharmacokinetic barriers in their clinical drug application. To minimize these undesirable drug properties, while maintaining the drug therapeutic activity, the prodrug approach was developed by covalently linking the active drug to a chemical moiety thus offering the utmost flexibility and at the same time enhancing the drug efficacy. The concept of a prodrug was first introduced by Albert in the late 1950s [1] to show that inactive chemical derivatives can be used to modify the physicochemical properties of drugs, in a short manner, in order to enhance their application and reduce associated side effects. The prodrug approach gained a lot of attention in the 1970s, as a powerful method for enhancing drug therapy. The universally accepted definition for a prodrug is a pharmacologically inert chemical drug that can be converted *in vivo* to the active drug molecule enzymatically or non-enzymatically while retaining its therapeutic effect. It is also worth noting that despite the fact that prodrugs and anologs take on similar structures, there are still some inherent differences (Figure 1). For example, prodrugs have reversible linkages whereas analogs lack bioreversibility.

Therapeutic drugs for cardiovascular diseases development soared because scientists were able to investigate complex molecular interactions that occur in the onset of disease and overcome pharmacological barriers by adapting to the prodrug approach. In turn, the amalgamation of these two developments provided a way to identify genetic alterations and to screen for a wider range of new drugs. The development of cardiovascular therapy experienced a rise in cardiac surgery, interventional diagnostics and therapeutics, noninvasive imaging, clinical electrophysiology, and genetic evaluations over the past few decades. Similarly, a huge advancement in implantable devices occurred leading to the invention of drug eluting stents (DES), where 80 % of major cardiac events were reduced one year post-surgery[3]. DES are devices implanted in patients with coronary artery disease, which release therapeutic drugs to inhibit restenosis. One of the major challenges with DES is developing effective therapeutic delivery systems capable of providing sustained and controlled release of the bioactive “agents” or “drugs”. 

The first most crucial step in the drug development process is to properly recognize and validate the drug target for the therapeutic application. Since prodrugs undergo a chemical reaction to form the parent drug once inside the body, this makes them very effective in controlling the release of a variety of compounds to targeted site and overcome barriers such as poor solubility, permeability and resistance to fast degradation. 

Although only one review [4] was found that discusses the role of prodrugs in cardiovascular therapy, it is important to highlight that in the last seven years produgs have affected the cardiovascular field in a very positive way by enhancing oral bioavailability by more than 50 % from the parent drug. Therefore in this review, we will discuss the extensive research efforts that have contributed towards the development of various types of prodrugs to treat cardiovascular diseases such as vascular thrombosis, hypertension, pulmonary hypertension, and atherosclerosis. Examples of newly developed techniques that have merged with the prodrug approach to enhance drug delivery by providing more controlled release and target specificity will also be described. 

## I. Prodrugs for the Treatment of Cardiovascular Diseases:

### I.1. Prodrugs for Vascular Thrombosis

#### I.1.a. Antithrombin produgs

**Figure 2:**
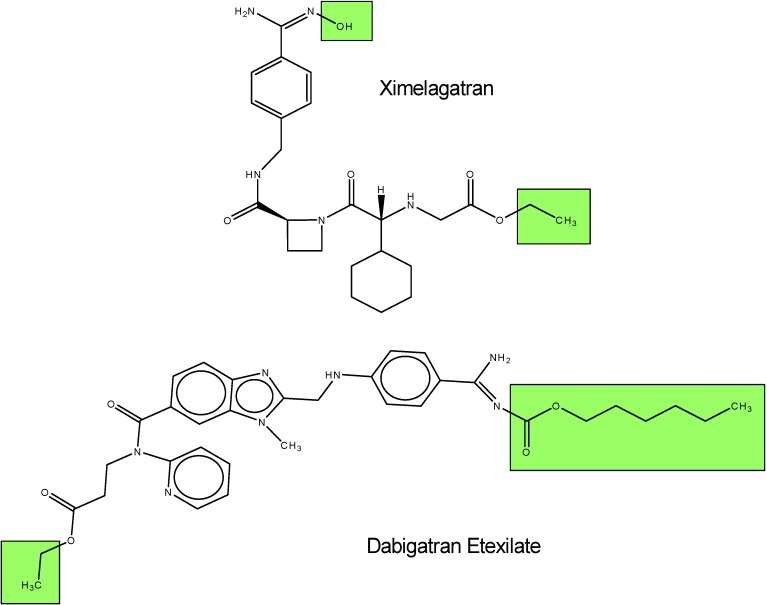
Chemical structures of Ximelagatran and Dabigatran Etexilate [11] .

Venous thromboembolism is a joint term for deep vein thrombosis and pulmonary embolism, where a blood clot is formed in a vein leading to major organs. Thrombin plays an intricate role in the development of venous thromboembolism; for that reason scientists were instigated to formulate drugs that can block thrombin formation. Platelets get promoted at the site of vascular injury by thrombin and fibrinogens which are converted to fibrins to offer stability for the surrounding platelets.*[5]* To date, in clinical development there are two oral direct prodrugs ximelagatran and dabigatran etexilate (BIBR 1048) that can directly inhibit thrombin. Ximelagatran (ethyl-2-[[(1*R*)-1-cyclohexyl-2-[(2*S*)-2-[[4-(N'-hydroxycarbamimidoyl)phenyl]methylcarbamoyl]-azetidin-1-yl]-2-oxo-ethyl]amino]acetate) is quickly taken up in the gastrointestinal tract and biologically converted to melagatran (N-[(1R)-2-[(2S)-2-[[[[4(aminoiminomethyl)phenylmethylaminocarbonyl]-1-azetidinyl]-1-cyclohexyl-2-oxoethyl-glycine]. Melagatran is known to inhibit the production [6] and the activity of human α-thrombin with a high binding affinity [7]. There are several differences between ximelagatran and melagatran. One ximelagatran contains an ethyl group at the carboxylic end and a hydroxyl group at the amidine end (Figure 2). Furthermore, at physiological pH melagatran is highly charged, whereas ximelagatran is inert and favors more lipopholic environments. Melagatran can be broken down further in the body, therefore it is removed via the renal route [8]. As for dabigatran etexilate (ethyl-3-{[(2-{[(4-{*N*'-[(hexyloxycarbonyl]carbamimidoyl}phenylaminomethyl}-1-methyl-1H-benzimidazol-5-yl)-carbonyl]-(2-pyridinylamino}propanoate), it is also rapidly absorbed after oral administration and converts to dabigatran (BIBR 953, N-[[2-[[[4-(aminoiminomethyl)phenyl]amino]methyl]-1-methyl-1*H*-benz-imidazol-5-yl]carbonyl]-*N*-2-pyridinyl*-β*-alanine), which is potent and selective for inhibiting thrombin. Recent studies have shown that dabigatran etexilate is very efficient in inhibiting thrombin in venous thrombosis models in rabbits [9] and rats [10], although it is worth noting that on a weight basis, experimental results showed that dabigatran had a greater potency than melagatran in a rat animal model study. 

#### I.1.b. Antiplatelet prodrugs

**Figure 3:**
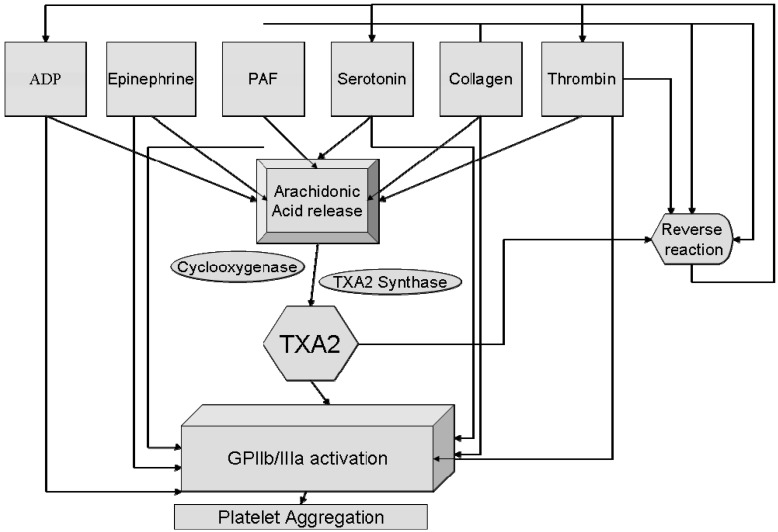
Various pathways for platelets activation. All the pathways lead to a common end point the GP IIb/IIIa receptors. (PAF: platelet activating factor, TXA_2_: thromboxane A_2_) (adapted from [14]).

The association of glycoprotein (GP) IIb/IIIa receptors with primary binding ligand fibrinogen induces platelet aggregation, which in turn promote coronary thrombosis in patients with unstable coronary artery disease [12, 13]. Figure 3 shows the various pathways that can lead to platelet aggregation. Clinical investigations have aimed at producing GP IIb/IIIa receptor inhibitors to block platelet aggregation. Some examples of effective GP IIb/IIIa receptor inhibitors are the monoclonal antibody c7E3 (abciximab, C_6462_H_9964_N_1690_O_2049_S_48_), epitifibatide (*N*-6-(aminoiminomethyl)-*N*-2-(3-mercapto-1-oxopropyl-L-lysylglycyl-L-α-aspartyl-L-tryptophyl-L-prolyl-L-cysteinamide) and tirofiban ((2*S*)-2-(butylsulfonylamino)-3-[4-[4-(4-piperidyl)butoxy]phenylpropanoic acid). Although these inhibitor drugs are effective in lowering platelet aggregation, they can only be administered intravenously and cannot be given orally. 

To overcome this limitation, an oral active prodrug was developed by Boehringer Ingelheim, under the name of lefradafiban [methyl 5-((((4'(imino(methoxycarbonyl)amino)-methyl)(1,1'-biphenyl)-4-yl)oxy)methyl)-2-oxo-pyrrolidineacetate] which is converted into an inhibitor drug, fradafiban [5-((((4'-aminoiminomethyl)-(1,1'-biphenyl)-4-yl)oxy)methyl)-2-oxo-3-pyrrolidineacetic acid] in the digestive system. A clinical pharmacology phase I study revealed that lefradafiban was able to inhibit platelet aggregation successfully *ex vivo* [15]. A phase II study was followed to address the dose treatment needed to provide 80 % inhibition of platelet aggregation in patients with stable coronary artery disease [16]. A dose dependent boost in fradafiban availability in plasma was observed when administering various amount of lefradafiban. It was found that the suitable dose for achieving 80 % inhibition required 45 mg of lefradafiban three times daily with patients undergoing percutaneous transluminal coronary angioplasty (PTCA). A greater amount of inhibition was observed in patients who received heparin and aspirin in addition to lefradafiban. Table 1 provides a list of prodrugs that were developed to stop platelet aggregation. The top three prodrugs displayed major side effects and in some cases, long term studies showed that the prodrugs were equivalent to aspirin, therefore any further studies were halted. As for the last prodrug, roxifiban (methyl 3-[[2-[(5R)-3-[4-(aminoimino-methyl)phenyl]-4,5-dihydro-5-isoxazolyl]acetyl]amino]-*N*-(butoxycarbonyl)-L-alanine) is the only drug from the list at present undergoing clinical investigations. Almost all prodrugs of this class undergo some degrees of thrombocytopenia, where it increases the incidence of bleeding. 

**Table 1:** A list of prodrugs orally administrated that inhibit platelet aggregation.

Prodrug	Type	Active Drug	% of Platelet Inhibition
Xemilofiban (4-pentynoic acid, 3-[[4-[[4-aminoimin-omethyl)phenyl] amino]-1,4 dioxobutyl]amino]-, ethyl ester, (3*S*))	Nonpeptidic	SC-54701A	50 %
Orbofiban ( *β*-alanine, *N*-[[[(3*S*)-1-[4-(aminoimino methyl)phenyl]-2-oxo-3-pyrrolidinyl]amino]carbonyl], ethyl ester)	Ethyl ester	SC-57101B	20.5 %
Sibrafiban (acetic acid, 2-[[1-[(2S)-2-[[4-[(Z)-amino-(hydroxy-imino)methyl] benzoyl]amino]-1-oxo-propyl]-4-piperidinyl]oxy], ethyl ester )	Nonpeptidic	Ro 44-3888	70-80 %
Roxifiban	Isoxazolinylacetamide and an ester	XV459	40-50 %

Treatment with roxifiban causes some phasic or activation changes of important platelet receptors [17]. Mousa *et al.* have found that a combination therapy of roxifiban with short-acting anticoagulant drugs like, for example, direct thrombin inhibitors (DTIs), lowers the risk of a patient to develop heparin-induced thrombocytopenia (HIT) [18]. Free and platelet bound drug-dependant antibodies (DDAb’s) are linked to be the cause of thrombocytopenia with roxifiban [19]. Recently, Barett *et al.* developed two early screening kits based on an ELISA assay that allows for the detection of DDAb in a patient [19]. By offering the patient proper dosing of roxifiban, the risk of bleeding or thrombocytopenia can be reduced. Interestingly, a dramatic improvement was observed no matter which DDAb type assay was used in this study, dropping the thrombocytopenia rate from 2% to < 0.2 % [19].

**Figure 4:**
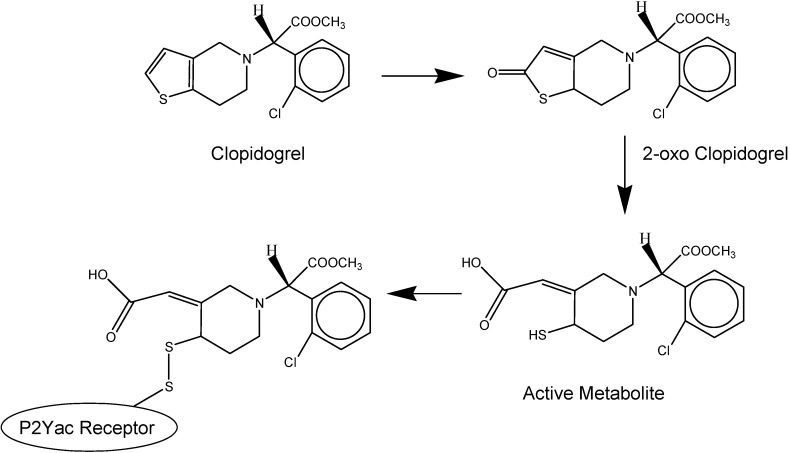
The mechanism of clopidogrel converting to its active metabolite in the liver (adapted from [23]).

After percutaneous coronary intervention (PCI), coronary stenting is performed to reduce restenosis. Followed coronary stenting, patients are given an antiplatelet drug clopidogrel which has shown superior efficacy in comparison to aspirin to inhibit stent thrombosis.*[20]* Clopidogrel ((+)-(*S*)-methyl-2-(2-chlorophenyl)-2-(6,7-dihydrothieno[3,2-c]pyridin-5(4*H*)-yl)acetate) is an inactive prodrug that is oxidized in the liver by cytochrome P_450_ to 2-oxoclopidogrel, leading to an active acid derivative metabolite (Figure 4). The acid derivative of clopidogrel releases its antiplatelet effect by generating a disulfide bond with the platelet P2Yac adenosine diphosphate (ADP) receptors [21,22,23,24] (Figure 4). Platelet aggregation is interceded by P2Yac (P2Y12) ADP receptor by blocking adenyl cyclase [24]. 

**Figure 5:**
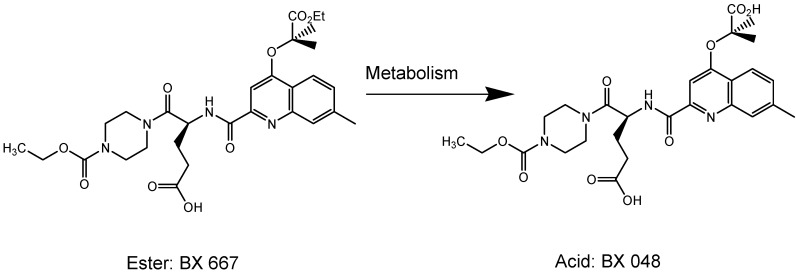
Chemical structure of BX667 and BX048 (adapted from [27]).

Heitzer *et al.* recently reported that in patients with symptomatic coronary disease that were given clopidogrel, this not only helped to reduce platelet aggregation but also enhanced endothelial function and nitric oxide (NO) bioavailabity, lowered oxidative stress and inflammatory response [25]. Clopidogrel, when combined with atorvastatin ([*R*-(R^*^,R^*^)]-2-(4-fluorophenyl)-β,δ-di-hydroxy-5-(1-methylethyl)-3-phenyl-4-[(phenylamino)carbonyl]-1*H*-pyrrole-1-heptanoic acid), which is a drug used to treat hypercholesteremia, loses its antiplatelet drug efficacy. This occurs because atorvastatin is also metabolized by cytochrome P_450_ and interferes with clopidogrel activation [26]. These results raise concerns and stress on the importance for patients to receive a point-of-care platelet function test when taking additional medications along with clopidogrel. Some limitations faced with clopidogrel are long period (>4 days) to achieve steady-state levels in order to inhibit platelets, narrow therapeutic index and high doses cause undesirable bleeding. Wang *et al*. proposed a new orally active reversible P2Y_12_ receptor antagonist BX 667 ((*S*)-4-({4-[1-(ethoxycarbonyl)-1-methylethoxy]-7-methyl-2-quinolyl}-carbamoyl)-5-[4-(ethoxycarbonyl)piperazin-1-yl]-5-oxopentanoic acid), which has the potential to overcome all the deficiencies noted with clopidogrel [27]. BX 667is metabolized in the liver to give the acid derivative BX 048 ((*S*)-4-({[4-(1-carboxy-1-methylethoxy)-7-methylquinolin-2-yl]-carbonyl}amino)-5-[4-(ethoxycarbonyl)piperazin-1-yl]-5-oxopentanoic acid (Figure 5), in the same way as clopidogrel. *In vivo *studies, demonstrated that BX 667 has a wider therapeutic index than clopidogrel in animal models suffering from thrombosis [27].

### I.2. Hypertension Prodrugs

**Figure 6:**
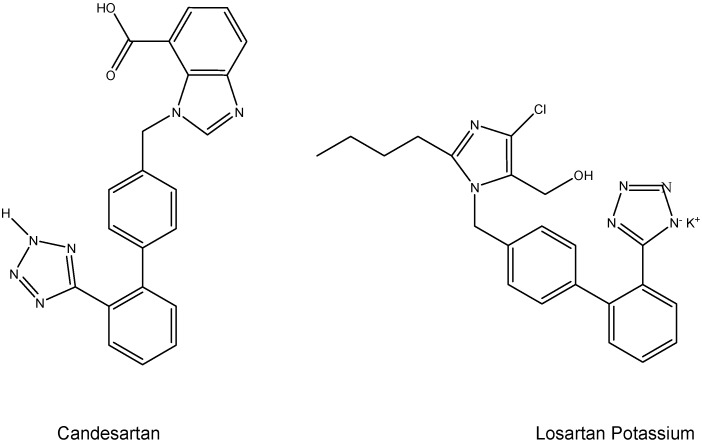
Chemical structures of ARB: candesartan (left) and losartan potassium (right). (BPT: biphenyltetrazole) [32].

Emerging pharmacological prodrugs for treating hypertension have revolutionized the treatment of mild to moderate hypertension [28,29,30]. Angiotensin II receptor blockers (ARB) regulate blood pressure, fluids and electrolyte balance with no adherent side effects. There are two prodrugs available: losartan potassium (1-((2'-(2*H*-tetrazol-5-yl)biphenyl-4-yl)methyl)-2-butyl-4-chloro-1*H*-imidazol-5-yl)methanol) and candesartan (3-((2'-(2*H*-tetrazol-5-yl)biphenyl-4-yl)methyl)-2-ethoxy-3*H*-benzo[d]-imidazole-4-carboxylic acid, Figure 6). After conversion to EXP 3174 (2-*n*-butyl-4-chloro-1-[(2'-(1*H*-tetrazol-5-yl)biphenyl-4-yl)methyl] imidazole-5-carboxylic acid) in the liver, they inhibit the effects of angiotensin II AT1 receptors selectively at the site of the receptor, but they do not block the AT2 receptors which mediate outcomes such as antiproliferation, vasodilation and apoptosis [31]. Most ARB contain biphenyl-tetrazole rings and only differ by their side chain. Recent clinical comparative study showed that candesartan lowered blood pressure considerably in comparison to losartan and was able to persist for a longer period of time [32]. 

**Figure 7:**
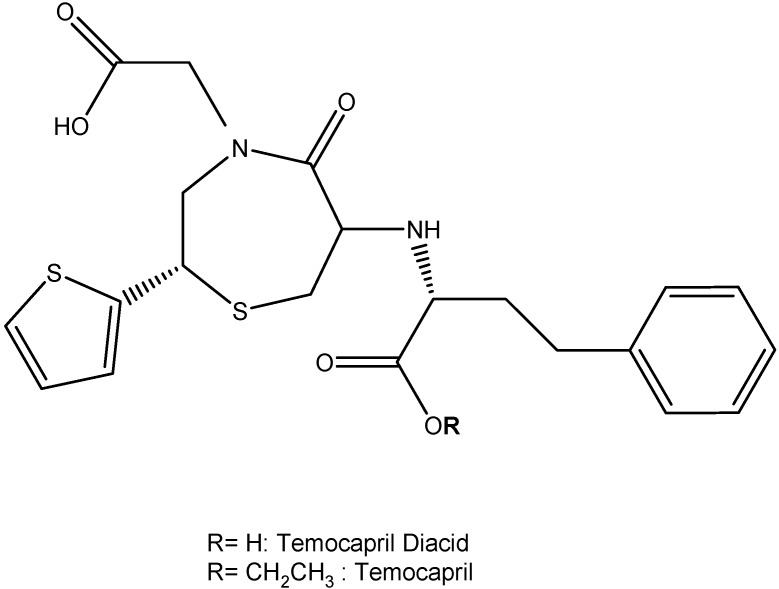
Chemical structure of temocapril diacid and temocapril [34].

Another class of drugs that control hypertension is angiotensin-converting enzyme inhibitors (ACEI). Not only they are used to treat hypertension, but they can also be used to treat congestive heart failure, myocardial infarctions and diabetic nephropathy. Temocapril (2-[(2*S*)-6-[[(2*S*)-1-ethoxy-1-oxo-4-phenylbutan-2-yl]amino]-5-oxo-2-thiophen-2-yl-1,4-thiazepan-4-yl]acetic acid, Figure 7), an ACEI prodrug, can convert more quickly into its diacids after administration then other ACEI prodrugs [33]. Since temocapril has shown great success with animal studies, human studies were conducted with patients suffering from hypertension and found it to be effective in lowering blood pressure and temocapril is currently available to the public in Japan [34]. It is thought that the success of temocapril can be attributed to its unique ability to act as a scavenger for oxygen free radicals [35]. Recently, a study by Kim *et al.* reported that the combination of temocapril with olmesartan medoxomil (CS-866, 5-methyl-2-oxo-1,3-dioxol-4-yl)methyl-5-(2-hydroxypropan-2-yl)-2-propyl-3-[[4-[2-(2H-tetrazol-5-yl)phenyl]phenylmethyl]imidazole-4-carboxylate), an angiotensin II AT_1_ receptor inhibitor prodrug, prevented intimal thickening after balloon angioplasty [35]. *In vivo*, olmesartan medoxomil can also quickly covert to an active acid metabolite RNH-6720 by de-esterification [36]. The conversion process generally occurs in the gut and is not dependent on cytochrome P_450_. The enhanced suppression of vascular smooth muscle cell proliferation is speculated to be mediated with either activated PDGF-β receptor or bradykinin or NO [35]. 

### I.3. Pulmonary Hypertension Prodrugs

Current treatments for pulmonary hypertension, i.e. when the lungs experience an increase in blood pressure, involve vasodilators and anticoagulants drugs that can inhibit cell proliferation and platelet aggregation [37]. Progressive pulmonary hypertension is defined as the buildup of collagen in pulmonary arteries which end up promoting fibroproliferation. Available drugs such as β-amino-propionitrile (C_3_H_6_N_2_) and D-penicillamine ((2*S*)-2-amino-3-methyl-3-sulfanylbutanoic acid) can inhibit collagen proliferation, but are toxic with long term use and also can inadvertently inhibit elastogenesis [38]. Proline analogue drugs are restricted due to high excretion rates and toxic side effects [38]. *cis*-4-Hydroxy-L-proline is linked via a lysine residue to poly(ethylene glycol) (CHOP-PEG) in order to lower its toxicity and promote its retention in blood vessels undergoing fibrosis. CHOP-PEG is a prodrug that inhibits fibrosis by suppressing the transforming growth factor-β/Smad signaling pathway [38]. In a study using a rat model with pulmonary hypertension induced by hypoxia, the CHOP-PEG had a 2x10^2 ^fold rate of fibrosis inhibition than monomeric CHOP while allowing the drug to be released in a dose dependant manner.*[39]* These preliminary data warrants further investigation of CHOP-PEG as a potential therapeutic agent for pulmonary hypertension and other diseases linked to fibroproliferation.

### I.4. Atherosclerosis Prodrugs

Atherosclerosis is a term used to describe an artery that has been hardened due to multiple formation of plaque that is generated by plasma proteins such as lipoproteins which are responsible to transport cholesterol and triglycerides. It has been shown by clinical and animal studies that estrogen can inhibit the occurrence of coronary atherosclerosis by blocking angiotensin-converting enzymes [40, 41]. Also an added benefit of estrogen is that it elevates the production of endothelium-derived NO which reduces vascular smooth muscle cell (VSMC) proliferation and leukocyte adhesions [42, 43]. Estrogen replacement therapies for postmenopausal women were contested because of discouraging clinical studies that showed poor cardiovascular protection and increase risk of breast cancer [44]. 

**Figure 8:**
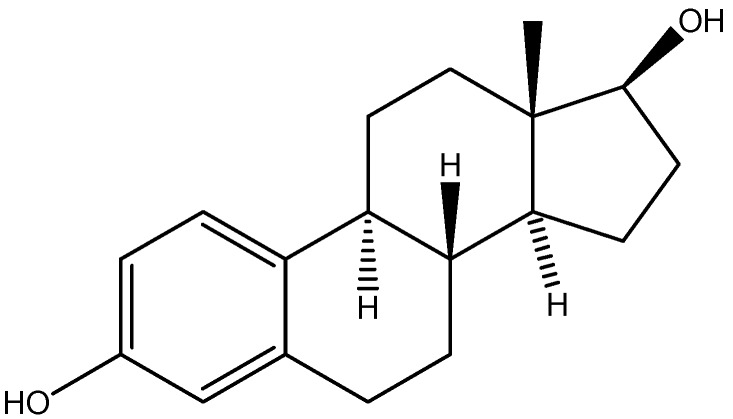
Chemical structure of 17-β-estradiol.

**Figure 9:**
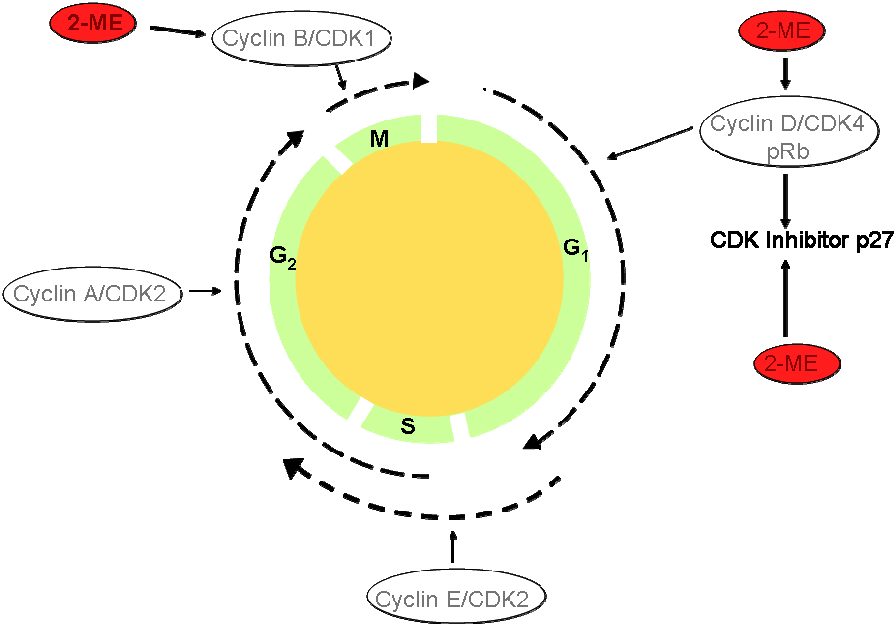
The eukaryotic cell cycle (adapted from [54]).

17-β-Estradiol (8*R*,9*S*,13*S*,14*S*,17*S*)-13-methyl-6,7,8,9,11,12,14,15,16,17-decahydrocyclopenta[a]-phenanthrene-3,17-diol, Figure 8), which is the most common estrogen hormone found in humans, has been linked to stop the initiation and the development of atherosclerosis in animal models [45]. Once inside the body, 17-β-estradiol is metabolized to 2-methoxyestradiol (2-ME) through methylation. 2-Methoxyestradiol can block rat and human VSMC migration and proliferation in spite of the mediator that is involved in controlling these processes with no affinity towards estrogen receptors [46]. The first *in vivo* evidence that 2-methoxyestradiol can guard against atherosclerosis was presented by Barchiesi *et al. *[47], who not only demonstrated the efficacy of 2-methoxyestradiol, but also provided an explanation for the involvement of 2-methoxyestradiol in the intracellular signaling pathway. The authors proposed that 2-methoxyestradiol inhibits cell division by blocking the expression and activation of cyclin and cyclin-dependent kinases (Cyclin-D1/cdk4), the expression of cdk inhibitor p27, tubulin polymerization and the expression of cyclooxygenase-2 (Figure 9). While Barchiesi *et al.* provided a detailed understanding of the processes involved in reducing vascular hardening, there is still no satisfactory explanation on what triggers 2-methoxyestradiol to initiate these processes. Overall, the work by Barchiesi and co-workers has provided solid expectation that 2-methoxyestradiol could be a suitable drug for the treatment of cardiovascular diseases for women without increasing the risk of breast cancer and also has the potential for therapeutic use in men too.

Most recently, 2-hydroxyestradiol (2OHE) was discovered as a prodrug of 2-methoxyestradiol with poor affinity towards estrogen receptors [48]. The 2-hydroxyestradiol conversion process is catalyzed by catechol-*O*-methyltransferase (COMT), which is an enzyme present in high levels in erythrocytes [49]. Generally, 2-hydroxyestradiol inhibits proliferation of smooth muscle cells (SMCs) in cells, but in studies where COMT was removed, the inhibition did not occur [50]. This further proves that COMT does mediate the conversion of 2-hydroxyestradiol to 2-methoxyestradiol. Apart from blocking SMCs growth, 2-hydroxyestradiol has been found to guard against puromycin aminonucleoside-induced nephropathy [51], monocrotaline-induced pulmonary hypertension [52] and angiotensin II-induced renal and cardiovascular injury [53]. *In vivo* studies with male rats demonstrated that the conversion of 2-hydroxyestradiol to 2-methoxyestradiol is very efficient and rapid, suggesting that the administration of 2-hydroxyestradiol is bioequivalent to the administration of 2-methoxyestradiol [48]. From a pharmacological therapeutic perspective, having 2-hydroxyestradiol bioequivalent to 2‑methoxyestradiol is advantageous because 2-hydroxyestradiol is chemically less stable and is easily formulated into a drug, as opposed to 2-methoxyestradiol. Zacharia *et al. *[48] have found that the methylation of 2-hydroxyestradiol occurs at a greater rate in coronary than in aortic VSMCs and endothelial cells (ECs). In aortic VSMCs, the formation of 2-methoxyestradiol is blocked by catecholamines, which are found in higher amounts in aortic cells then coronary cells. Also COMT is not only responsible for mediating the production of 2-methoxyestradiol, but also for the breakdown of catecholamine. 

A potential alternative approach for improving estradiol delivery was presented by Zovko *et al. *[55] where they bound poly(α,β-(*N*-2-hydroxyethyl-DL-aspartamide))-poly(α,β-(*N*-2-aminoethyl-DL-aspartamide)) copolymer (PAHA) to estradiol-3-benzoate (EB). By conjugating the drug to the polymer, they expected to increase the estradiol solubility, prolong its release and increase stability. To date, there has not been any report that demonstrates the clinical efficacy of PAHA-EB. Recent findings by Tanguay and co-workers [56] presented a debatable notion on whether direct local delivery of a drug at the injured site is more efficient than the produg approach for combating restenosis following PTCA. In their study, a single dose of 17-β-estradiol was administered locally at the time of stent implantation with porcine models suffering from coronary arterial injury. After introduction of 17-β-estradiol, all pigs experienced a reduction in SMCs growth compared to the control model, lower amount of inflammation and improved vascular reendothelialization after 28 days post-stenting procedure [56]. These results demonstrate the potential of direct local delivery of 17-β-estradiol in treating and preventing neointimal hyperplasia and late stent thrombosis.

## II. Delivery Strategies

It is vital to design drug delivery systems where the carrier can actively release its drug specifically inside the diseased tissue. Andresen *et al. *[57] introduced secretory phospholipase A_2 _as a site-specific trigger to prodrug loaded liposomes for the treatment of cancerous tissue. Another interesting development by Brioschi *et al. *[58] describe the application of solid lipid nanoparticles as effective drug vehicles for treating brain tumors. Recently, human mesenchymal stem cells from adipose tissue were introduced as delivery vehicles for site-specific enzyme prodrug conversion approach for chemotherapy [59]. Although these different prodrug delivery strategies described above allowed better control of drug distribution, metabolism, and elimination, they serve only for the treatment of cancer type diseases. There is a small amount of literature that reports new delivery strategies that provides specific and controlled release of prodrugs for the treatment of cardiovascular diseases as described below. 

### II.1. Antibody Targeted, Triggered, Electrically Modified Prodrug-Type Strategy (ATTEMPTS)

A method that unites the prodrug approach with targeting specificity, where the drug is inactive during transport and then active when released to specific tissue targets without any toxic affects would be very effective in the field of drug delivery. Based on this theory a two-step approach called ADEPT (the antibody-directed enzyme prodrug therapy) was developed and showed good results in delivering small drugs to target sites [60,61,62,63]. A major drawback with the ADEPT approach is that it limits its application to small drugs only because the prodrug can only be conjugated chemically and released by enzyme cleavage. Macromolecular drugs like proteins or enzymes are not suitable for the ADEPT approach because they contain several functional groups which can make the conjugation of the drug non-selective. 

**Figure 10:**
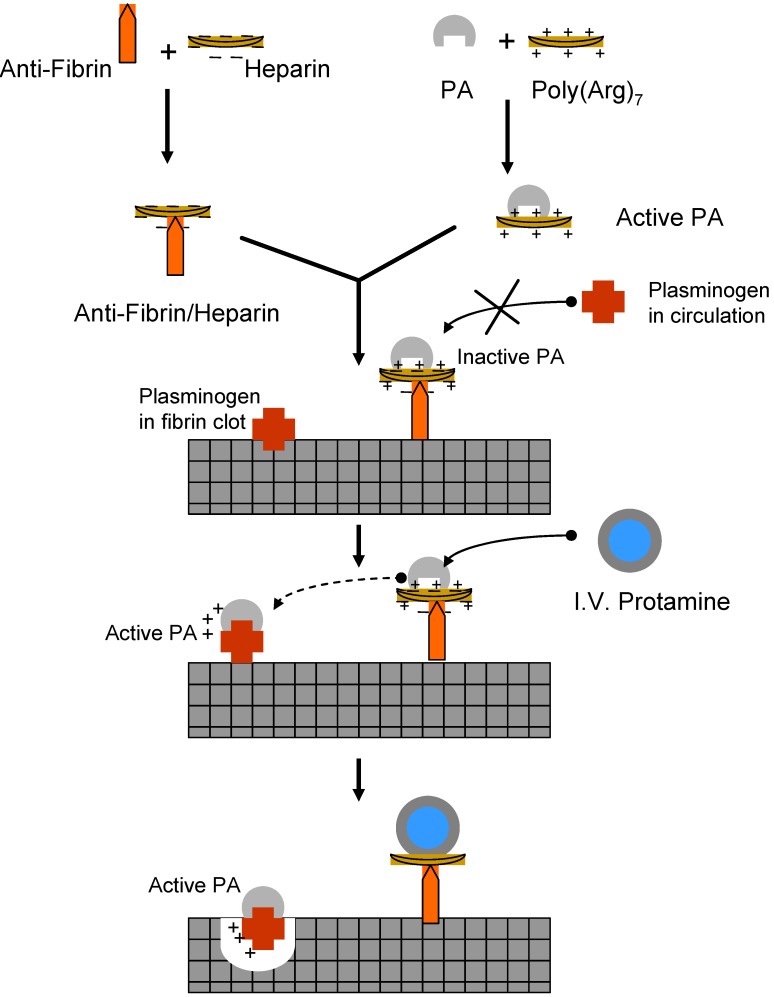
Schematic diagram of the ATTEMPTS approach (adapted from [64]).

**Figure 11:**
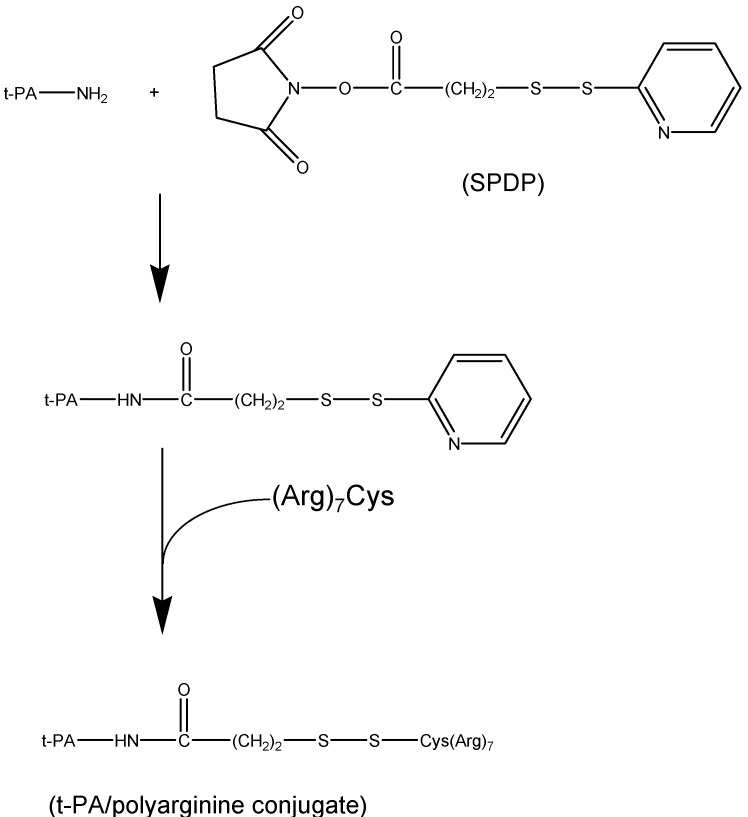
The synthesis of modified t-PA with t-PA and poly((Arg)_7_) using *N*-succinimidyl-3-2-pyridyldithio propionate (SPDP) (adapted from the manuscript by Liang *et al.* [64]).

A heparin/protamine-based prodrug system was developed to control the delivery of macromolecular thrombolytic agents such as tissue-type plasminogen activator (tPA). The desirable effect of enzyme-based drugs is accredited to their high specificity and efficiency to their target analyte. The so-called **a**ntibody **t**argeted, **t**riggered, **e**lectrically **m**odified **p**rodrug-**t**ype **s**trategy (ATTEMPTS), is aimed to allow antibody-directed administration of inactive t-PA without toxic side effects. Model drugs that were adapted to the ATTEMPTS approach are Azure-A-modified trypsin, peptide (Arg)_7_Cys-modified t-PA (Figure 11) and Immunoglobulin G (IgG)-59D8 antifibrin antibody-t-PA. 

**Figure 12:**
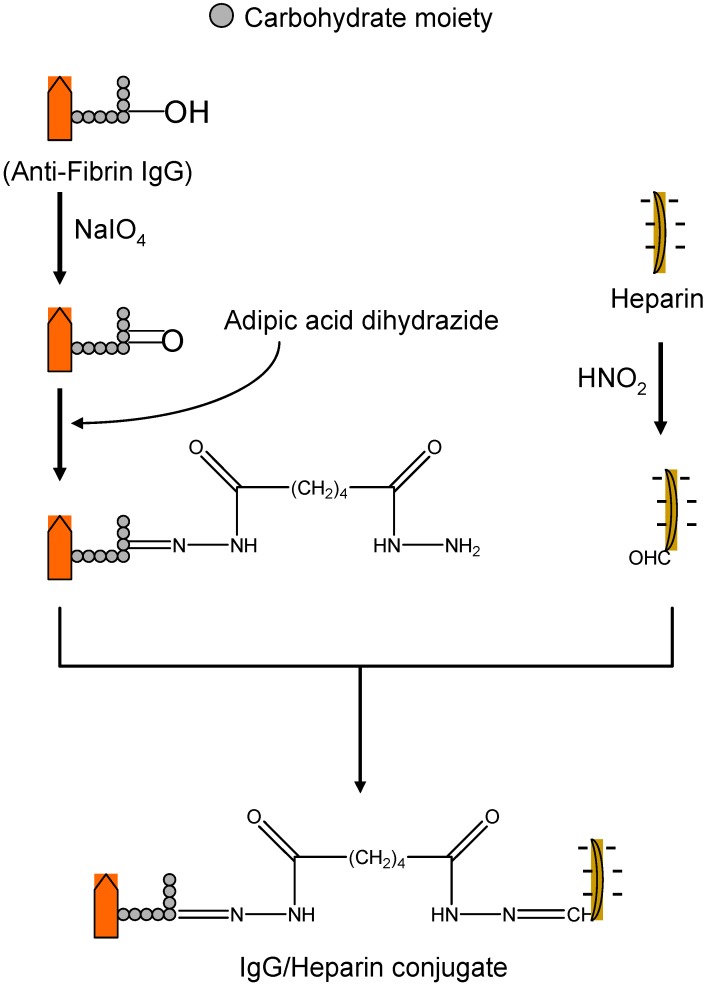
The synthesis of the heparin-antifibrin IgG conjugate (adapted from [66]).

The ATTEMPTS method has more moderate constraints, as such, the modified enzyme binding strength must be more robust than the heparin and the antithrombin (III) so that the enzyme can stay coupled to and be inhibited by the heparin. Another stipulation is that the enzyme binding affinity must be weaker than protamine in order for the protamine to activate its release from heparin inhibition. For instance, the selection of IgG-59D8 antifibrin antibody was based on its high selectivity towards fibrin than other existing clot-targeting antibodies. The feasibility to conjugate heparin to IgG-59 was challenging and required the development of a site directed coupling method (Figure 12) that permitted end-point attachment of the heparin [64]. In general, most studies showed that nor the cation nor recombinant DNA modification of t-PA has affected the activation of plasminogen or the binding ability towards fibrin and its catalytic activity [64]. All prodrugs of t-PA showed higher affinity towards heparin or heparin-antifibrin then the native drug [64]. 

Recently, Yang *et al.* in a rat thrombosis model study demonstrated the effectiveness of the ATTEMPTS approach for delivering t-PA successfully without any bleeding side-effects [65]. Cation modified t-PA (CM-t-PA) coupled to heparin antibody conjugate was administered intraveneously. Then protamine was also introduced intraveneously after a specific time period to trigger the release of CM-t-PA and resulted in a lower clot weight than the control.

### II.2. Prodrug Approach Combined with Layer by Layer Assembly

As mentioned earlier, a major obstacle in designing advanced drug formulations for cardiovascular implants like DES is the ability to provide controlled release of the bioactive agent. The advantages of having controlled or sustained drug release are greater efficacy, minimal toxicity, and improved patient convenience over conventional methods. Layer by Layer (LBL) assembly is quite a simple process based on the electrostatic interactions of two or more oppositely charged macromolecules, often referred to as polyelectrolytes, which yield multilayer structures ranging in thickness from tens to hundreds of nanometers. It is important for the bioactive coating on the DES to be able to reduce platelet adhesion and blood coagulation. A copious amount of literature have showed that coating with LBL films containing serum albumin [67,68,69,70], heparin [67, 68, 71, 72], dextran [73, 74], and chitosan [71, 73, 74, 75] improve the resistance of blood coagulation.

**Figure 13:**
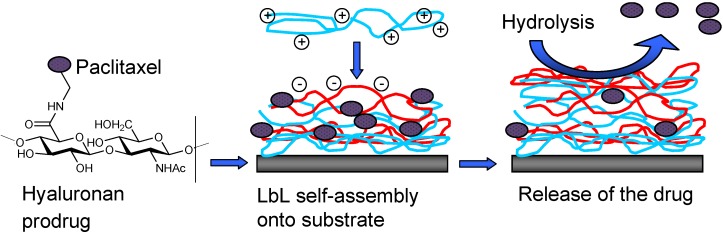
A schematic representation of the LBL process using paclitaxel-hyaluronic acid and chitosan [78].

A multilayer film of chitosan (CH) and hyaluronic acid (HA) was assembled on the surface of a stent in the presence of sodium nitroprusside [sodium pentacyanonitrosylferrate(III), NO donor] by Thierry *et al*. [76]. The availability of NO enhanced vascular tone and wall dynamics of the artery and served to block platelet adhesions and SMCs proliferation. At the same time the polymers hyaluronic acid and chitosan have anti-inflammatory and wound-healing properties that serve to help inhibit neointimal hyperplasia. Additionally, in an *in vitro* study, Thierry *et al.* coated the interior of a damaged artery with hyaluronic acid and chitosan loaded with arginine and observed 91 % reduction in platelet adhesion [77]. A few years later, the authors reported a novel approach by combining the delivery of hydrophobic drugs via a macromolecular prodrug approach with LBL assembled functionalized multilayers of hyaluronic acid and chitosan [78]. A hyaluronic acid ester prodrug of the chemotherapeutic agent paclitaxel (1*S*,2*S*,3*R*,4*S*,7*R*,9*S*,10*S*,12*R*,15*S*)-4,12-diacetoxy-15-{[(2*R*,3*S*)-3-(benzoylamino)-2-hydroxy-3-phenylpropanoyl]oxy}-1,9-dihydroxy-10,14,17,17-tetramethyl-11-oxo-6-oxatetracyclo[11.3.1.0~3,10~.0~4,7~]heptadec-13-en-2-yl benzoate) was synthesized (Figure 13). Release of paclitaxel from the loaded multilayers upon hydrolysis of the ester linkage was gradual. The LBL approach can be used to deposit various drugs onto the stent surface at the same time providing controlled release of the drug. 

## Conclusions

It is clear from the foregoing, that the prodrug approach promises to be a resourceful technique to treat cardiovascular diseases. One of the main advantages, it offers site specificity and chemical stability. The formation of vascular thrombosis is mainly induced by platelet aggregation. But the molecular events that occur to induce platelet aggregation require the activation of GP IIb/IIIa receptors and by no means, it is a simple process because many factors can act as inducers. Through surveying a plethora of prodrugs that treat vascular thrombosis, it was evident from the *in vivo* studies that most platelet reducing prodrugs performed better when given in conjunction with other drugs such as aspirin, heparin and DTIs. This is not surprising because most prodrugs that reduce thrombosis experience side effects such as thrombocytopenia. Only one prodrug has been shown to lower the risk of bleeding along with platelet aggregation and that is BX667 in rat and dog models, although more clinical testing is needed to warrant its potential for human application. An important point was raised with the clopidogrel prodrugs that if patients are given additional drugs to treat other ailments, numerous times these drugs would interfere with the activation of the prodrug leading to unsatisfactory patient recovery. Therefore, it is important for patients to receive point-of-care platelet function test when combining other drugs along with clopidogrel.

Treatment for mild to moderate hypertension using ACEI prodrugs hold more promise than using ARB type prodrugs. The ACEI prodrug temocapril has not yet reached the North American market but in Japan it is readily available and has shown to be excellent in lowering blood pressure. Also, the combination of temocapril with CS-866 an ARB prodrug was very effective in minimizing intimal thickening after balloon angioplasty. Most available anti-fibrotic drugs for advanced cases of pulmonary hypertension are very toxic, the development of prodrugs like CHOP-PEG not only provided better blockage of fibrosis but also overcame toxicity and allowed for controlled release of drug. 

It is important to bear in mind that most cardiovascular related diseases are generated by multiple contributing factors. For instance, atherosclerosis is not only caused by VSMC proliferation, but also by the buildup of lipids and fibrous elements [79]. Since 2-methoxyestradiol inhibits VSMC proliferation efficiently, it does not automatically mean that 2-methoxyestradiol stops the atherosclerotic process adequately to eliminate completely any cardiovascular risk. Therefore, it is evident that diseases like atherosclerosis require a multi-therapeutic approach. Nonetheless, this does not dismiss the potential for synergizing 2-methoxyestradiol producing type prodrugs with other therapies such as ACEI’s or ARB to rectify completely the treatment of atherosclerosis. 

One of the greatest challenges in the development of advanced drug formulations is the capability of delivery systems to provide target specificity and sustained release of the bioactive drug without side effects. The ATTEMPTS approach showed that by adapting the prodrug approach with antibodies/heparin complexes, a drug can be delivered effectively to a specific target site without any bleeding side effects. Additionally, the blending of the prodrug approach with the LBL assembly methodology on DES have revolutionized the delivery of hydrophobic drugs like paclitaxel allowing for gradual release of drug while the bioactive polymers (i.e. HA and CH) reduced neointimal hyperplasia. Only a few articles in the literature reports enhanced delivery systems for cardiovascular prodrugs, whereas the bulk of this area of research is focusing on cancer inhibiting type prodrugs. Therefore, in the future, we hope to see more scientists make more advances with cardiovascular prodrug enhanced delivery approaches.

Most prodrugs today that are available for treating cardiovascular diseases are designed to be administered either orally (most common) or intravenously. Although, in some cases like during cardiovascular implant surgery, direct administration of the drug on the injured site or having it on the implant itself has shown to enhance the drug efficacy. The future of cardiovascular therapy with prodrugs looks very promising and combination therapy of prodrugs would be the key that unlocks the door of recovery for many patients. 
